# Supplementary material for: Low HDL Cholesterol Efflux Capacity Indicates a Fatal Course of COVID-19
Source: Antioxidants (Basel). 2022 Sep 21;11(10):1858. doi: 10.3390/antiox11101858 (PMC9598682; doi:10.3390/antiox11101858)
Supplement: Supplementary file 1 [file antioxidants-11-01858-s001.zip › antioxidants-1906143-supplementary.pdf]

**Low HDL cholesterol efflux capacity indicates a fatal course of COVID-19**

## Supplementary Material

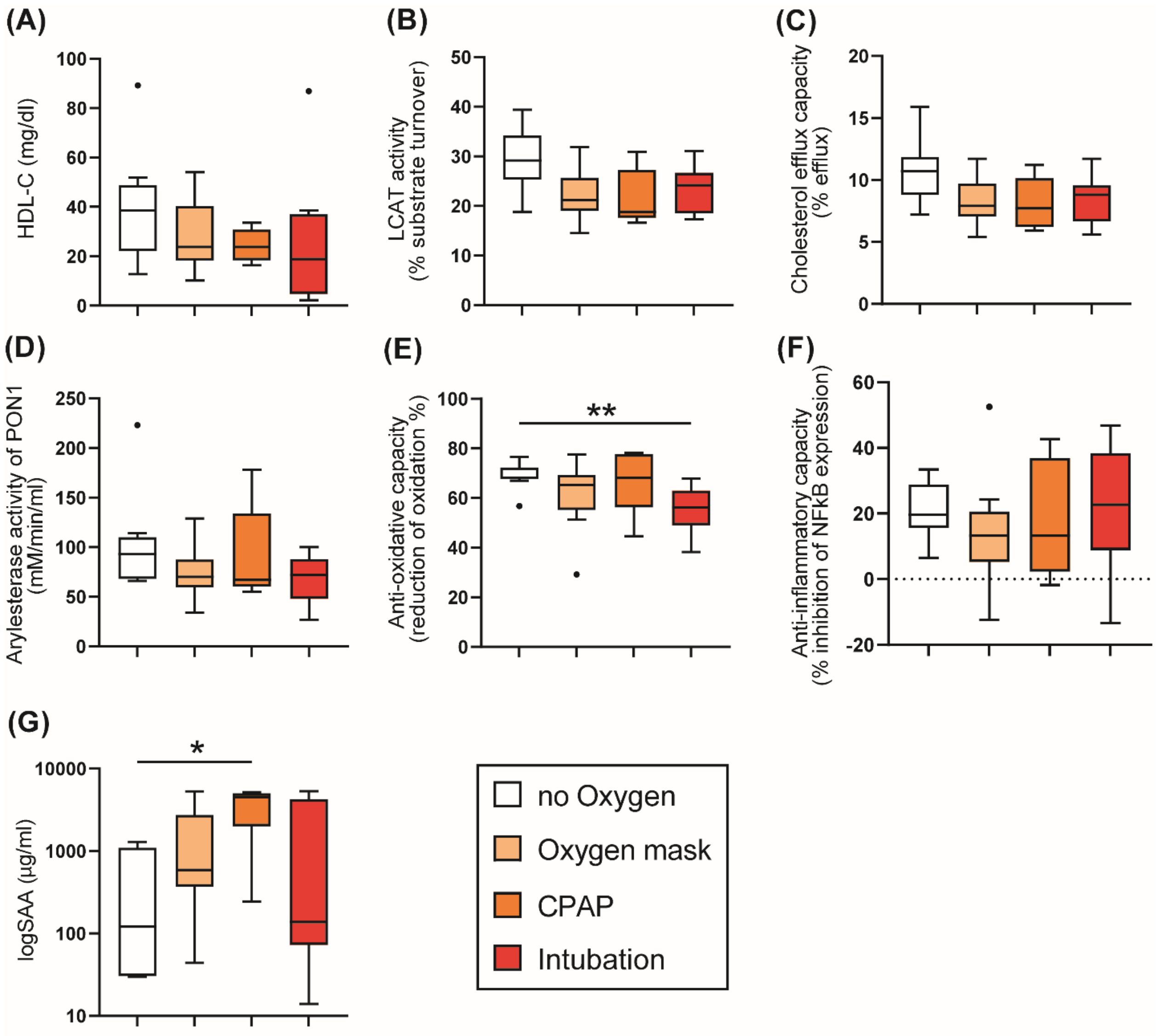


**Supplementary Figure S1.** HDL-related parameters and COVID-19 patients receiving no or different types of oxygen treatment. **(A)** HDL-cholesterol levels (mg/l); **(B)** LCAT activity (% substrate turnover); **(C)** Cholesterol efflux capacity (%); **(D)** arylesterase activity (mM/min/ml serum); **(E)** anti-oxidative capacity (% reduction of oxidation); **(F)** anti-inflammatory capacity (% inhibition of NFκB expression) ; **(G)** logSAA (μg/ml). Multiple comparisons were calculated with Kruskal-Wallis test. *$p<0.05$, **$p<0.01$.
